# Relative Handgrip Strength Diminishes the Negative Effects of Excess Adiposity on Dependence in Older Adults: A Moderation Analysis

**DOI:** 10.3390/jcm9041152

**Published:** 2020-04-17

**Authors:** Robinson Ramírez-Vélez, Miguel Ángel Pérez-Sousa, Antonio García-Hermoso, Fabrício Zambom-Ferraresi, Nicolás Martínez-Velilla, Mikel L. Sáez de Asteasu, Carlos A. Cano-Gutiérrez, David Rincón-Pabón, Mikel Izquierdo

**Affiliations:** 1 Department of Health Sciences, Public University of Navarra, Navarrabiomed-Biomedical Research Centre, IDISNA-Navarra’s Health Research Institute, C/irunlarrea 3, Complejo Hospitalario de Navarra, 31008 Pamplona, Navarra, Spain; antonio.garciah@unavarra.es (A.G.-H.); fabriciogigante@hotmail.com (F.Z.-F.); nicolas.martinez.velilla@navarra.es (N.M.-V.); mikel.lopez.saezdeasteasu@gmail.com (M.L.S.d.A.); mikel.izquierdo@gmail.com (M.I.); 2 Faculty of Sport Sciences, University of Huelva, Avenida de las Fuerzas Armadas s/n 21007, 21004 Huelva, Spain; perezsousa@gmail.com; 3 Laboratorio de Ciencias de la Actividad Física, el Deporte y la Salud, Facultad de Ciencias Médicas, Universidad de Santiago de Chile, USACH, Santiago 7500618, Chile; 4 CIBER of Frailty and Healthy Aging (CIBERFES), Instituto de Salud Carlos III, Pamplona, 28001 Navarra, Spain; 5 Hospital Universitario San Ignacio-Aging Institute, Pontificia Universidad Javeriana, Bogotá 110111, Colombia; ccano@javeriana.edu.co; 6 ZIPATEFI (Zona de Investigaciones de Posgrados, Terapia Respiratoria y Fisioterapia de Areandina), Fundación Universitaria del Área Andina, Pereira 110231, Colombia; nicheunal@hotmail.com

**Keywords:** fat mass, obesity, muscle strength, physical function, functional dependence

## Abstract

The adverse effects of fat mass on functional dependence might be attenuated or worsened, depending on the level of muscular strength. The aim of this study was to determine (i) the detrimental effect of excess adiposity on dependence in activities of daily living (ADL), and (ii) whether relative handgrip strength (HGS) moderates the adverse effect of excess adiposity on dependence, and to provide the threshold of relative HGS from which the adverse effect could be improved or worsened. A total of 4169 participants (69.3 ± 7.0 years old) from 244 municipalities were selected following a multistage area probability sampling design. Measurements included anthropometric/adiposity markers (weight, height, body mass index, waist circumference, and waist-to-height ratio (WHtR)), HGS, sarcopenia “proxy” (calf circumference), and ADL (Barthel Index scale). Moderation analyses were performed to identify associations between the independent variable (WHtR) and outcomes (dependence), as well as to determine whether relative HGS moderates the relationship between excess adiposity and dependence. The present study demonstrated that (i) the adverse effect of having a higher WHtR level on dependence in ADL was moderated by relative HGS, and (ii) two moderation thresholds of relative HGS were estimated: 0.35, below which the adverse effect of WHtR levels on dependency is aggravated, and 0.62, above which the adverse effect of fat on dependency could be improved. Because muscular strength represents a critically important and modifiable predictor of ADL, and the increase in adiposity is inherent in aging, our results underscore the importance of an optimal level of relative HGS in the older adult population.

## 1. Introduction

Muscle strength and mass decline with aging [1]. The importance of preserving optimal muscle strength in middle- and older-age adults has been recently highlighted in epidemiological studies showing that muscle strength is an important predictor for all-cause [2] and cancer [3] mortality. Physical function in older adults declines with the loss of skeletal muscle [1,4], and a recent study reported that a non-weak handgrip strength (HGS) level (cut-off points ranged from 17.4 to 8.6 in men and 10.1 to 4.9 in women) is related to decreased odds of intrinsic capacity impairments (i.e., the interaction between the physical and mental capacity of an individual) among older adults [5].

Several studies have indicated that the aging process produces a series of changes in body composition, usually without affecting the body mass index or weight, but producing an accumulation of fat as individuals get older [6,7]. Beyond its corresponding effect on health, excess adiposity has a harmful impact on muscle quality and quantity [8]. Consequently, the convergence of aging and fat mass may create a perfect storm for skeletal muscle catabolism [9] and a decline in physical function [10].

Handgrip strength (HGS) is the most common index of muscle strength, owing to its ease of assessment, low cost, and simplicity, and it is considered a valid “proxy” of overall muscle strength for clinical and epidemiological studies [11]. Lower HGS correlates strongly with cardiovascular disease [2] and mortality [12], and several studies [13,14] have highlighted its protective role against activities of daily living (ADL) dependence in older adults. Thus, maintaining an optimum HGS is an effective determinant factor for healthy aging [5,15]. In this context, several studies have shown that aging is associated with a decline in handgrip strength, and several studies have highlighted the fact that an increase in fat mass contributes to a deterioration of HGS in older adults [8,16]. These aforementioned processes can be viewed as a cascade of events, beginning with aging, which are associated with greater muscle fat infiltration [17,18]. Aging and accumulation of infiltrating fat leads to a decline in muscle quality and quantity—therefore resulting in a poorer performance (lower muscle strength)—and, ultimately, affecting functional dependence in ADL [19,20].

This worsening of muscle strength can be explained several biological factors. First, fat infiltration induces changes in contractile function [21] in the different manifestations of strength (isometric, concentric, and eccentric) [22,23]. Second, aging and fat infiltration coexist in an environment marked by a loss of muscle strength and power, also known as dynapenia [24], which is related to a reduction in central activation, a decrease in motor unit number and size, and an alteration in the excitation–contraction cycle [25]. Finally, aging and dynapenia are related to a greater presence of proinflammatory activity, which seem to be responsible for the deterioration of muscular function (fat infiltration into muscle), and visceral fat increases and subcutaneous fat decreases with aging [26]. We therefore hypothesized that muscle strength could play a preventive role in this association.

The adverse effects of abdominal obesity on functional dependence, might be attenuated or worsened depending on the level of muscular strength. Additionally, relative handgrip strength is associated with functional dependence. Thus, central adiposity may have an effect between dependence status and relative handgrip strength after potential confounding variables such as age, gender, and/or lifestyle. Accordingly, describing the magnitude of these risk factors in older adults could be important for prioritizing prevention and public health efforts. Nevertheless, to our knowledge, no studies have examined the moderator role of muscle strength based on HGS between excess of central adiposity and functional dependence.

The aim of the present study was two-fold: (i) to examine the detrimental effect of abdominal obesity on functional dependence in ADL, and (ii) to discern whether relative HGS moderates the adverse effect of abdominal obesity on dependence, as well as to provide the threshold of relative HGS from which the adverse effect could be improved or worsened.

## 2. Materials and Methods

### 2.1. Study Design and Sample Population

The data for this secondary cross-sectional study were obtained from the Health and Well-being and Aging Survey in Colombia 2015 (SABE, from initials in Spanish: Salud, Bienestar y Envejecimiento, 2015), a multicenter project conducted from 2014 to 2015 by (in Spanish: Ministerio de Salud y la Protección Social de Colombia) [27]. The study included the Colombian population aged ≥60 years, and the indicators were disaggregated by age ranges, sex, ethnicity, and socioeconomic level.

A total of 23,694 surveys were conducted at the national level. A total of 6530 segments were planned to obtain the surveys (4928 urban and 1602 rural), with an expected average of 4.7 adults per segment. The standardized process for each home visit involved the identification of the participants, the registration of the demographic data, the signing of the informed consent, the application of the established filters and the selection criteria, the signing of assent when necessary, and the completion of the questionnaire by the interviewer. For this subsample, the calculation of the sample size was carried out, taking into account national representation. A total of 86 municipalities were selected, including the four large cities. For this analysis, we used data from 4169 participants included as a subsample with HGS measures. The rationale and detailed methodology of the SABE Colombia has been described in another document [28].

Institutional review boards involved in developing the SABE 2015 study (University of Caldas, ID protocol CBCS-021-14, and University of Valle, ID protocol 09-014 and O11-015) reviewed and approved the study protocol. The study protocol for the secondary analysis was approved by the Human Subjects Committee at the Pontificia Universidad Javeriana (ID protocol 20/2017-2017/180, FM-CIE-0459-17) in accordance with the Declaration of Helsinki of the World Medical Association and Resolution 8430 of 1993 of the then Ministry of Health of Colombia on technical, scientific, and administrative standards for conducting research with humans. All participants provided written informed consent.

### 2.2. Measurements

Data collection staff were trained by the research teams of the coordinating centers (University of Caldas, and University of Valle, from Colombia) for face-to-face interviews and physical measurements. Anthropometry measurements included height and body weight, which were measured with a portable stadiometer (SECA 213, Hamburg, Germany) and an electronic scale (Kendall graduated platform scale), respectively. Body mass index (BMI) was calculated in kilograms per square meter from the measured body weight and height. Waist circumference (WC) was measured over the midpoint between the lower border of the ribs and iliac crest in the midaxillary plane, at the end of normal expiration. The waist-to-height ratio (WHtR) was measured as the ratio of the waist circumference (in cm) to the height (in cm). We used WHtR as a measure for abdominal obesity because is a useful tool in clinical practice and has been shown to be a reliable parameter for predicting whole-body fat percentage and visceral adipose tissue [28]. The calf circumference was used for screening sarcopenia because it is a reliable, easy, and low-cost tool in clinical practice [4]. Following the recommendation of the WHO Expert Committee [29] and Rolland et al. [4], a cut-off of calf circumference ≤31 cm was considered as sarcopenia. HGS, including absolute and relative—HGS (kg)/body mass (kg)—were assessed with a Takei dynamometer (T.K.K., Takei Scientific Instruments Co., Ltd., Niigata, Japan), including the highest value (kg) from two attempts (both hands). This allowed us to be more accurate when comparing older adults with different body sizes and to focus on muscle quality rather than muscle quantity. The coefficients of variation for body weight, height, waist circumference, calf circumference, and HGS were 23.2%, 6.5%, 12.2%, 11.2%, and 42.2%, respectively.

Nutritional status was evaluated through Mini-Nutritional Assessment extended version [30]. Functional impairment was assessed with an ADL evaluation using a Spanish-adapted version of the physical level ADL (Barthel Index), recommended for epidemiological studies in older adults [31]. The Barthel Index scores are in multiples of five, ranging from 0 (completely dependent) to 100 (independent in basic). The Barthel index scores are classified as follows: 100 means independence, 91–99 low-level dependency, 75–90 mild dependency, 50–74 moderate dependency, 25–49 severe dependency, and 0–24 total dependency [32].

For lifestyle characteristics, personal habits regarding alcohol intake (participants were categorized as those who do not drink and those who drink less than 1 day per week, 2 to 6 days a week, or every day) and cigarette smoking (participants were categorized as those who do not smoke and those who have never-smoked, those who currently smoke, or those who previously smoked) were recorded. A “proxy physical activity” report was conducted by the following questions: (i) “Have you regularly exercised, such as jogging or dancing, or performed rigorous physical activity at least three times a week for the past year?”; (ii) “Walk, at least three times a week, between 9 and 20 blocks (0.6 to 1.2 km) without resting?”; (iii) “Walk, at least three times a week, eight blocks (0.5 km) without resting?”. Participants were considered physically active if they responded affirmatively to two of the three questions. Medical information including multimorbidity, as well as chronic conditions adapted from the original SABE study, were assessed by asking the participants if they had been diagnosed, by a physician, with hypertension, type 2 diabetes mellitus, chronic obstructive pulmonary disease, cardiovascular diseases (heart attack, angina), stroke, cancer, arthritis, osteoporosis, or sensory impairments (vision and hearing loss). Medication use was evaluated with the question “do you currently take or use any prescription medication?”.

Race/ethnicity grouped as indigenous (people belonging to various indigenous groups, such as Ika, Kankuamo, Emberá, Misak, Nasa, Wayuu, Awuá, Mokane), black “mulato” or Afro-Colombian, white, and others (mestizo, gypsy, etc.) was assessed by self-reporting. Socioeconomic status (SES) was determined on the basis of the housing stratum (1 to 6), with level 1 being the highest poverty and level 6 the highest wealth. This classification is a measure developed by the National Government of Colombia that considers physical characteristics of the dwellings and their surroundings. The classification in any of the six strata approximated the hierarchical socioeconomic difference from poverty to wealth and vice versa.

### 2.3. Statistical Analysis

Descriptive analyses using mean ± standard deviation (SD) for the continuous variables and frequency distribution for categorical variables were used to obtain the characteristics of the sample. The normality of the data was examined by the Kolmogorov–Smirnoff test. Significant differences between men and women were analyzed using Student’s *t*-test or the chi-square (χ^2^) post-hoc test.

The PROCESS macro in the SPSS statistical software package, version 24.0 (IBM, Chicago, IL, USA) for Windows, was used to conduct a moderation analysis. Preliminary analysis showed no significant interactions between gender and abdominal obesity in relation to functional dependence (*p* = 0.814); therefore, all analyses were performed with men and women together. Moderation analysis was conducted to examine whether WHtR levels were related to increased dependence and to determine whether this negative effect was moderated by relative HGS. This relationship used ordinary least squares regression analysis when predicting continuous variables (WHtR and relative HGS in the study). A simple slope plot was used to visualize the effect of the moderator. The Johnson–Neyman approach was used to test the point in which the relative HGS value moderated the relationship between WHtR levels and dependence. The Johnson–Neyman technique determined, along a continuum of moderator values (relative HGS), the region of significance on the relationship between the independent and dependent variables [33]. All tests were adjusted for sex, age, alcohol, smoking status, and physical activity habits.

## 3. Results

Of the 4169 study participants, 56.2% were female and 43.8% were male (Table 1). Anthropometric data, including BMI, waist circumference, and WHtR, described the principal characteristics of the sample, such as overweight or obesity and an excess of fat mass. The differences between sex for these variables were significant (*p* < 0.05), with the exception of calf circumference. Regarding performance outcomes—computed from absolute HGS and relative HGS—men showed a significantly higher performance than women. Additionally, the ethnic distribution was dissimilar between sexes, except for the Afro-Colombian ethnic group. We observed a major proportion of white and other ethnic groups (mestizo, gypsy, etc.). A major proportion of participants were found to be in SES level 2 and significant differences were observed between sexes in all SES levels except level 1.

Likewise, there were significant differences between sexes regarding lifestyle habits including smoking, alcohol consumption, and physical activity “proxy” recommendations. According to self-report comorbidities presented by participants, there was a prevalence of visual problems (57.7%) and high blood pressure (53.7%) in both sexes. Regarding the distribution difference between males and females, we found significant differences in hearing problems, high blood pressure, type 2 diabetes mellitus, arthritis, and osteoporosis. In addition, there were significant differences in medication use and nutritional status. Finally, the prevalence of dependency was 8.1% and 4.3% for mild and moderate dependency, respectively. However, when we combined all three levels of dependency (mild, moderate, and severe) this rose to 12.5%, with 522 older adults dependent in ADL.

**Figure 1:**
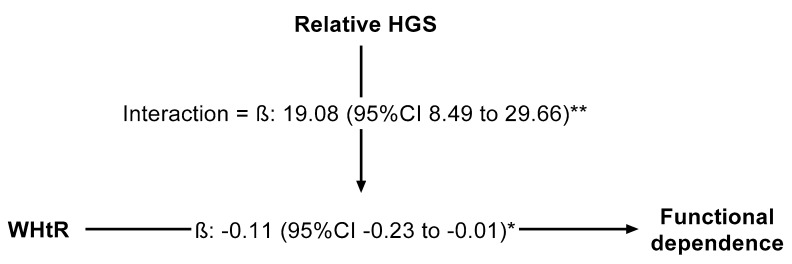
Moderation models. Beta expressed as unstandardized regression coefficients and 95% confidence interval. Because there was substantial covariance between strength capacity and body mass—and, moreover, the links between muscle strength and both physical function and chronic health were mediated by the proportion of strength relative to body mass—grip strength (HGS) was relative as strength per body mass (i.e., (HGS in kilograms)/(body mass in kilograms)). Moderation analysis in which relative handgrip strength moderate the relationship between waist-to-height ratio (WHtR) and functional dependence, adjusted by age, gender, and lifestyle (alcohol intake, smoking, and physical activity “proxy”); * *p* < 0.01; ** *p* < 0.001.

Figure 1 shows the results from the regression model, where it shows the moderation analysis based on ordinary least squares regression, in which there is an inverse relationship between the excess of adiposity, measured via WHtR, on functional dependence in older adults. This path known as direct effect (*β* = −0.11 (−0.23, −0.01)) was moderated by relative HGS. Therefore, the adverse effect of excess adiposity on functional dependence was moderated by relative HGS (*β* = 19.08 (8.49, 29.66)).

**Figure 2:**
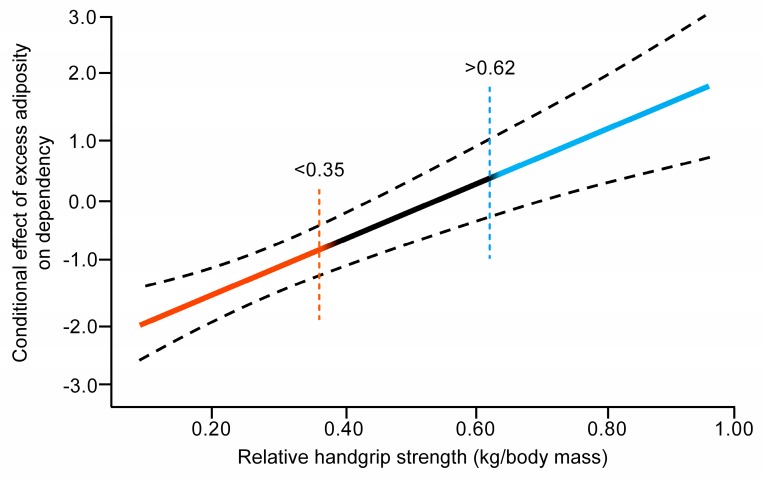
Regression slope estimate and 95% confidence intervals for the relationship between moderator variable (relative HGS) and adverse effect of WHtR levels on dependency level in activities of daily living (ADL), based on the Johnson–Neyman procedure. Red line indicates negative region of significance at moderator value (<0.35 of relative HGS). Blue line indicates the positive region of significance at moderator value (>0.62 of relative HGS). Black line represents neutral region of significance.

To elucidate a possible estimate point from which the moderator value has a moderator effect, the Johnson–Neyman statistical approach was used. The result is shown in Figure 2. The slope shows the continuum of the moderator (relative HGS expressed as kilogram per kilogram of body weight) and the different regions of significance. The first region was found to be less than 0.35, denoting that the adverse effect of excess adiposity, based on WHtR, on dependence could be aggravated for those in this region. Secondly, a significant positive region was found from 0.62, indicating that the adverse effect of WHtR could be ameliorated for those who were above this point. Lastly, a “black” region was observed, which indicated that the adverse effect did not improve or worsen in those with an HGS between the lower and upper thresholds.

## 4. Discussion

The present study investigated the moderator role of HGS on the adverse effect of WHtR on dependency in older Colombian adults. The major finding of the study was that the adverse effects of high WHtR levels on dependency were found to be moderated by relative HGS. Two moderation thresholds of relative HGS were estimated: 0.35, below which the adverse effect of WHtR levels on dependency was aggravated, and 0.62, above which the adverse effect of fat on dependency improved. Accordingly, our results indicated that older adults with higher WHtR could experience more dependence in ADL than older adults with lower WHtR; however, this unfavorable effect was moderated by relative HGS. Consequently, older adults with high relative HGS levels could attenuate the negative effect of adiposity. Therefore, age-related declines in muscle mass and strength are often detected by reductions in HGS.

The findings of the present study are supported by several previous studies. For example, de Carvalho et al. [8] found that abdominal obesity is associated with lower HGS, accelerating the decline of muscle strength. A possible explanation for this phenomenon is that excessive adiposity can downregulate the anabolic actions of testosterone [34], growth hormones [35], and insulin [36], which may contribute to a progressive loss of muscle mass and associated function in both sexes. Additionally, excessive adipose tissue can induce a proinflammatory state by the action of several cytokines (e.g., higher plasma concentrations of tumor necrosis factor-alpha and interleukin-6), which is associated with lower muscle strength [37] and disability in older adults [38].

No previous studies have reported a moderator role of HGS on the relationships studied here. We found that higher relative HGS could attenuate the adverse effect of abdominal obesity on functional dependence in older adults. Our findings show that higher central obesity has an adverse effect on functional independence. It is therefore likely that functional independence in ADL will be reduced in those older adults with abdominal obesity. However, this negative effect could be moderated by relative HGS. Consequently, the adverse effect may worsen, improve, or even disappear, depending on the relative HGS of older adults. Our findings indicate that muscle strength relative to body weight can play a crucial role between WHtR levels and dependency. Specifically, if older adults have a high WHtR value and a relative HGS above 0.62, the adverse effect on dependency could be mitigated or even disappear. Conversely, if older adults have a high WHtR value and a relative HGS below 0.35, it could worsen the adverse effect on dependency.

Biomechanical and neuromuscular scientific evidence could justify the moderator role of HGS between excess of central adiposity and functional dependence. For example, it is well reported that abdominal obesity is related to a greater body weight, and consequently walking more slowly might help to keep the dynamic balance between steps, as well as to maintain shorter the cadence and length of steps to optimize gait pattern [39]. Another plausible reason might be neuromuscular deterioration, as there is an association between obesity/high-fat mass content and poor muscle quality [23,40,41,42], with an impairment of force production relative to body weight [22,23]. Conversely, abdominal obesity may be linked to reduced HGS, as every 10 cm increase in WC has been shown to be associated with a 3.56 kg lower HGS in middle-aged and older men [24]. Additionally, every 1 kg increase in HGS for older women was associated with a 0.13 s decrease in the timed up-and-go test, 0.03 s decrease in 3 m walk time, and 1% decrease in chair rise time [43]. With regard to ADL, McGrath et al. [44] determined that high baseline grip strength decreased the odds ratio (OR) of developing disability in ADL (OR 0.95) and instrumental ADL (OR 0.92) among older Mexican Americans. These findings suggest that a minimum level of strength is a prerequisite for physical function and that, when strength is above the minimum required level, it may serve as reserve capacity, which is beneficial in preventing functional limitation in the future [45]. Accordingly, maintaining muscle strength is an important factor for maintaining function during the aging process [2,3]. Future research should expand upon the longitudinal associations between HGS and clinically relevant health outcomes that are mediated (e.g., in both instrumental activities and ADL) or moderated (e.g., obesity) by other factors [45].

The strengths of the study include the large population-based study in older, Latin-American adults. Additionally, we carried out complex statistical analyses to determine the role of muscle strength to circumvent the detrimental effect of excess adiposity on dependence. As well as this, through the Johnson–Neyman statistical approach, we provided two thresholds of relative HGS, which we believe will add to the knowledge base to improve clinical practice and exercise programs in this population.

There are some limitations of the study design that need to be considered. First, the cross-sectional design limits drawing any causal inferences. Second, the assessment of excess adiposity can result in bias because of the proxy method (i.e., WHtR levels), and therefore, standardized measures of body composition should be used. Third, the classification of dependency was based on a self-report questionnaire. Thus, we are unable to say whether low grip strength (with or without excess adiposity) leads to higher risk of neuromuscular/ADL abnormalities, or conversely, whether poor neuromuscular/dependency profiles lead to declines in grip strength (i.e., reverse causation). Future research is needed to better describe the age- and sex-specific trajectories of HGS as a predictor of comorbidities across the lifespan, and perhaps, just as importantly, to apply robust analyses that can compartmentalize risk into hierarchical categories. Finally, the thresholds for HGS are open to discussion and the values may vary depending on the comorbidities that individuals present with.

## 5. Conclusions

In summary, older adults with excess adiposity have major dependency in ADL. However, this adverse effect can be moderated by relative HGS. Our findings bring two thresholds of relative HGS as moderators of the adverse effect: <0.35, in which the adverse effect of abdominal obesity on dependence could worsen, and >0.62, in which the detrimental effect could be improved or even disappear. Because muscle strength represents a critically important and modifiable predictor of ADL [13,14,43], and an increase in body fat is inherent in aging [46], our results underscore the importance of an optimal level of relative HGS among the older adult population. Thus, this study provides support for the importance of considering both HGS and WHtR as contributors to diagnostic functional disability/dependence, and healthcare professionals should encourage participation in physical activity to improve muscular fitness in old age [47].

## Figures and Tables

**Table 1:** Characteristics of the study participants.

Characteristics	Men (*n* = 1825, 43.8%)	Women (*n* = 2344, 56.2%)	Overall (*n* = 4169)	*P* for Gender
**Anthropometric, mean ± SD**				
Age (years)	69.9 ± 7.2	68.9 ± 6.9	69.3 ± 7.0	<0.0001
Height (cm)	163.1 ± 6.7	151.1 ± 6.2	156.4 ± 8.7	<0.0001
Body weight (kg)	68.1 ± 11.8	63.3 ± 11.9	65.4 ± 12.1	<0.0001
BMI (kg/m^2^)	26.1 ± 3.9	28.3 ± 4.9	27.3 ± 4.6	<0.0001
Waist circumference (cm)	93.2 ± 10.7	91.6 ± 10.9	92.3 ± 10.8	<0.0001
Waist-to-height ratio	0.57 ± 0.1	0.60 ± 0.1	0.59 ± 0.1	<0.0001
Calf circumference (cm)	34.7 ± 3.3	34.7 ± 3.8	34.7 ± 3.6	0.807
**Functional performance, mean ± SD**				
Absolute HGS (kg)	27.5 ± 8.0	17.3 ± 5.3	21.8 ± 8.3	<0.0001
Relative HGS/body weight (kg/kg)	0.41 ± 0.1	0.27 ± 0.1	0.33 ± 0.1	<0.0001
**Race/ethnic group, *n* (%)**				
Indigenous	149 (9.1)	103 (5.0)	252 (6.8)	0.004
Black “mulato” or Afro-Colombian	173 (10.6)	181 (8.7)	354 (9.6)	0.671
White	478 (29.3)	696 (33.6)	1174 (31.7)	<0.0001
Others *	831 (51.0)	1092 (52.7)	1923 (51.9)	<0.0001
Missing	194	272	466	-
**Socioeconomic status, *n* (%)**				
Level I	689 (37.8)	752 (32.1)	1441 (34.6)	0.097
Level II	755 (41.4)	987 (42.1)	1742 (41.8)	<0.0001
Level III	345 (18.9)	511 (21.8)	856 (20.5)	<0.0001
Level IV	27 (1.5)	67 (2.9)	94 (2.3)	<0.0001
Level V–VI	9 (0.5)	27 (1.2)	36 (0.9)	0.003
**Lifestyle outcomes, *n* (%)**				
Alcohol intake	451 (24.7)	122 (5.2)	573 (13.7)	<0.0001
Smoking	287 (15.7)	171 (7.3)	458 (11.0)	<0.0001
Physical activity “proxy”	1375 (75.3)	1965 (83.8)	3340 (80.1)	<0.0001
**Multimorbidity/chronic conditions, *n* (%)**				
Hearing loss	492 (26.9)	463 (19.7)	955 (22.9)	<0.0001
Visual loss	1029 (56.3)	1378 (58.7)	2407 (57.7)	0.029
High blood pressure	844 (46.2)	1395 (59.5)	2239 (53.7)	<0.0001
Diabetes mellitus 2	258 (14.1)	410 (17.5)	668 (16.0)	0.004
Chronic pulmonary disease	168 (9.2)	244 (10.4)	412 (9.9)	0.195
Coronary heart disease	235 (12.8)	326 (13.9)	561 (13.4)	0.340
Stroke	73 (4.0)	78 (3.3)	151 (3.6)	0.267
Cancer	74 (4.1)	124 (5.3)	198 (4.7)	0.062
Arthritis	285 (15.6)	822 (35.1)	1107 (26.5)	<0.0001
Osteoporosis	82 (4.5)	378 (16.1)	460 (11.0)	<0.0001
**Medication use, *n* (%)**	451 (24.7)	122 (5.2)	573 (13.7)	<0.0001
**Nutritional status**				
Malnutrition	31 (1.9)	66 (3.2)	97 (2.7)	<0.0001
Risk of malnutrition	502 (31.3)	718 (35.0)	1220 (33.4)	<0.0001
Normal nutritional status	1073 (66.8)	1267 (61.8)	2340 (64.0)	<0.0001
Missing	219	293	512	-
**Functional dependence, *n* (%)**				
Severe dependency	0 (0.0)	3 (0.1)	3 (0.1)	-
Moderate dependency	68 (3.7)	113 (4.8)	181 (4.3)	<0.0001
Mild dependency	101 (5.5)	237 (10.1)	338 (8.1)	<0.0001
Non-dependency	1656 (90.7)	1991 (84.9)	3647 (87.5)	<0.0001

Data are presented as mean ± SD or number (percentage) of participants. Significant differences between the men and women groups were analyzed by Student’s *t*-test or χ^2^ test. BMI: body mass index. * Others (mestizo, gypsy, etc.).
